# Discerning suicide in drug intoxication deaths: Paucity and primacy of suicide notes and psychiatric history

**DOI:** 10.1371/journal.pone.0190200

**Published:** 2018-01-10

**Authors:** Ian R. H. Rockett, Eric D. Caine, Hilary S. Connery, Gail D’Onofrio, David J. Gunnell, Ted R. Miller, Kurt B. Nolte, Mark S. Kaplan, Nestor D. Kapusta, Christa L. Lilly, Lewis S. Nelson, Sandra L. Putnam, Steven Stack, Peeter Värnik, Lynn R. Webster, Haomiao Jia

**Affiliations:** 1 Department of Epidemiology, West Virginia University, Morgantown, West Virginia, United States of America; 2 Injury Control Research Center, West Virginia University, Morgantown, West Virginia, United States of America; 3 Department of Psychiatry, University of Rochester Medical Center, Rochester, New York, United States of America; 4 Injury Control Research Center for Suicide Prevention, University of Rochester Medical Center, Rochester, New York, United States of America; 5 Division of Alcohol and Drug Abuse, McLean Hospital, Boston, Massachusetts, United States of America; 6 Department of Psychiatry, Harvard Medical School, Boston, Massachusetts, United States of America; 7 Department of Emergency Medicine, Yale University School of Medicine, New Haven, Connecticut, United States of America; 8 School of Social and Community Medicine, University of Bristol, Bristol, United Kingdom; 9 Pacific Institute for Research and Evaluation, Calverton, Maryland, United States of America; 10 Curtin University, Perth, Australia; 11 Office of the Medical Investigator, University of New Mexico School of Medicine, Albuquerque, New Mexico, United States of America; 12 UCLA Luskin School of Public Affairs, Los Angeles, California, United States of America; 13 Department of Psychoanalysis and Psychotherapy, Medical University of Vienna, Vienna, Austria; 14 Department of Biostatistics, West Virginia University, Morgantown, West Virginia, United States of America; 15 Department of Emergency Medicine, Rutgers New Jersey Medical School, Newark, New Jersey, United States of America; 16 Department of Criminal Justice, Wayne State University, Detroit, Michigan, United States of America; 17 Department of Psychiatry and Behavioral Neuroscience, Wayne State University, Detroit, Michigan, United States of America; 18 Estonian-Swedish Mental Health and Suicidology Institute, Tallinn, Estonia; 19 PRA Health Sciences, Salt Lake City, Utah, United States of America; 20 Department of Biostatistics, Columbia University, New York, New York, United States of America; 21 School of Nursing, Columbia University, New York, New York, United States of America; University of Queensland, AUSTRALIA

## Abstract

**Objective:**

A paucity of corroborative psychological and psychiatric evidence may be inhibiting detection of drug intoxication suicides in the United States. We evaluated the relative importance of suicide notes and psychiatric history in the classification of suicide by drug intoxication versus firearm (gunshot wound) plus hanging/suffocation—the other two major, but overtly violent methods.

**Methods:**

This observational multilevel (individual/county), multivariable study employed a generalized linear mixed model (GLMM) to analyze pooled suicides and undetermined intent deaths, as possible suicides, among the population aged 15 years and older in the 17 states participating in the National Violent Death Reporting System throughout 2011–2013. The outcome measure was relative odds of suicide versus undetermined classification, adjusted for demographics, precipitating circumstances, and investigation characteristics.

**Results:**

A suicide note, prior suicide attempt, or affective disorder was documented in less than one-third of suicides and one-quarter of undetermined deaths. The prevalence gaps were larger among drug intoxication cases than gunshot/hanging cases. The latter were more likely than intoxication cases to be classified as suicide versus undetermined manner of death (adjusted odds ratio [OR], 41.14; 95% CI, 34.43–49.15), as were cases documenting a suicide note (OR, 33.90; 95% CI, 26.11–44.05), prior suicide attempt (OR, 2.42; 95% CI, 2.11–2.77), or depression (OR, 1.61; 95% CI, 1.38 to 1.88), or bipolar disorder (OR, 1.41; 95% CI, 1.10–1.81). Stratification by mechanism/cause intensified the association between a note and suicide classification for intoxication cases (OR, 45.43; 95% CI, 31.06–66.58). Prior suicide attempt (OR, 2.64; 95% CI, 2.19–3.18) and depression (OR, 1.48; 95% CI, 1.17–1.87) were associated with suicide classification in intoxication but not gunshot/hanging cases.

**Conclusions:**

Without psychological/psychiatric evidence contributing to manner of death classification, suicide by drug intoxication in the US is likely profoundly under-reported. Findings harbor adverse implications for surveillance, etiologic understanding, and prevention of suicides and drug deaths.

## Introduction

Following a 19% decline between 1986 and the turn of the century, the United States (US) suicide rate rose from 10.40 per 100,000 population in 2000 to 13.75 in 2015—a 32% increase [1]. Although large, this rise may be a serious underestimate due to increasing misclassification of intoxication suicides [2], a trend detected in other countries [3,4]. The US drug intoxication mortality rate for persons 15 years and older has risen by 257%—from 7.81 deaths per 100,000 in 2000 to 20.07 in 2015 [1]. This increase primarily implicates the component involving pharmaceutical opioids, heroin, and other illicit opioids [5,6]. Distinguishing manner of death showed that 67% of drug fatalities in the 15 and older population in 2000 were classified as accident, followed by suicide, undetermined intent (hereafter “undetermined”), and homicide at 19%, 14%, and 0.1% [1]. By 2015, accidents constituted 84% of these deaths versus 10% for suicide, 6% for undetermined, and 0.1% for homicide. At 322%, the 15-year rise in the accident component of the drug intoxication death rate was 9-fold higher than the corresponding rise in the drug intoxication suicide rate. Viewed in the context of under-resourced and overburdened emergency healthcare [7,8] and medicolegal death investigation systems [9,10], these differentials and discrepancies buttress a scenario of growing suicide misclassification. Compounding the situation, medical examiners and coroners need substantial affirmative evidence to assign suicide or homicide as the manner of death, whereas information can be scant for supporting an accident option, their major default [11].

Suicide is a highly stigmatized and perpetually undercounted phenomenon in the US [12]. Firearm (gunshot wound), hanging/suffocation, and drug intoxication are the leading methods, accounting for 88.3% of suicides in 2015 [1], with respective components of 49.8%, 26.8%, and 11.7%. However, strong corroborative psychological and psychiatric evidence, in the form of a suicide note and psychiatric history, may be much more important to ascertainment of suicide by drug intoxication than by the other two overtly violent methods [13–15].

A meta-analysis of 27 psychological autopsy studies [16] and a systematic review of 22 case-control studies and 54 case series [17] indicated that up to 90% of suicides in the US and other Western countries had a diagnosable psychiatric disorder at time of death. An American multiple-cause-of-death study showed that, whereas diagnosed psychiatric disorders distinguished suicides from deaths whose manner was classified as accident, they were documented in less than 10% of the death certificates for the suicides [18]. A subsequent American multiple-cause-of-death study of pooled suicides and undetermined deaths found that cases without psychiatric documentation manifested a 260% excess likelihood of an undetermined classification [19]. An English coroner study reported a large prevalence gap in suicide notes [20], as potentially pivotal evidence [21,22], between suicide and undetermined cases—50% versus 11%. Undetermined is universally regarded as the manner of death category most susceptible to suicide misclassification [23–27], and drug intoxication and other poisoning is the predominant injury cause/mechanism represented among undetermined cases in the US [19, 28]. In the United Kingdom, suicide rates are officially presented as a composite of rates for registered suicides and undetermined deaths [29], an accommodation that increases the suicide rate by approximately 20% [30].

In this multilevel (individual/county), multivariable study of the association between psychological/psychiatric documentation and suicide versus undetermined manner of death classification, based on microdata from the US National Violent Death Reporting System (NVDRS), we first calculated the prevalence of a documented affective disorder, suicide note, and past suicide attempt among suicide and undetermined cases. We then evaluated associations between these variables and suicide versus undetermined classification, and whether emergent associations were more paramount among drug intoxication cases than gunshot and hanging/suffocation cases. Our incorporation of undetermined deaths, as well as registered suicides, not only provided a window on the nature of suicide misclassification within the undetermined manner of death category [15], but within the accident category—as a much larger reservoir for obscuring drug intoxication suicides [31,32].

## Materials and methods

### National Violent Death Reporting System (NVDRS) Restricted Access Database

The principal data source for this study was the Restricted Access Database in the NVDRS, a state, territory, and incident-based surveillance system that employs public health informatics for making data linkages to produce detailed, individual-level information about suicide and other violent deaths [33]. Administered by the Centers for Disease Control and Prevention (CDC), the NVDRS primarily comprises data from death certificates, law enforcement records, and medical examiner and coroner records. This system variably includes such optional supplementary data as crime laboratory reports and hospital records. Its Restricted Access Database contains de-identified information that includes geographic location, circumstances, and personal sociodemographic characteristics. In this study, the microdata pertained to the 17 states, disaggregatable to county of death, which participated in the NVDRS throughout our observation period, 2011–2013. These states were Alaska, Colorado, Georgia, Kentucky, Maryland, Massachusetts, New Jersey, New Mexico, North Carolina, Ohio, Oklahoma, Oregon, Rhode Island, South Carolina, Utah, Virginia, and Wisconsin. Our study population comprised deaths from intentional self-harm, that is, registered suicides (ICD-10: U03, X60-X84, Y87.0), and undetermined deaths (ICD-10 Y10-Y34 and Y87.2, Y89.9) whose state and county of death were known. It was further limited to decedents aged 15 years and older, since less than 1% of known suicides nationally were younger [1]. Decedents in the study population totaled 40,581.

Table 1 provides comparative population and mortality data for the 17 NVDRS states in this study and for the entire US in 2012, the mid-year in our observation period. With a population approaching one-third of that of the US, these states closely resembled the nation in their age and sex composition, manner of death distribution, and crude and age-adjusted all-cause, suicide, and undetermined mortality rates. This demographic concordance supports the generalizability of our results, although NVDRS states overrepresented non-Hispanic Whites and Blacks and underrepresented Hispanics. Natural causes or diseases accounted for the overwhelming preponderance of deaths in both the NVDRS states and nationally. However, the disproportionate toll that suicide and other injury exact in premature mortality and truncated life expectancy [34–36] is masked in the equating of individual deaths in most conventional summary mortality measures.

**Table 1:** Selected population and mortality characteristics: 17 National Violent Death Reporting System (NVDRS) states and the United States, 2012.

Characteristic	NVDRS States	United States
Size	95,297,084	314,112,078
**Age (years)^a^**	**Percentage**^**a**^	
0–14	19.5%	19.5%
15–34	27.2	27.5
35–54	27.3	27.0
55–74	20.1	19.9
75+	5.9	6.1
**Sex^a^**		
male	49.1	49.2
**Race/ethnicity^a^**		
non-Hispanic White	69.0	63.9
non-Hispanic Black	15.3	12.9
Hispanic	10.4	16.9
Other	5.3	6.3
**Manner of Death^b^^,^^c^**		
homicide	0.62	0.67
suicide	1.66	1.62
accident	5.33	5.12
undetermined	0.23	0.19
natural causes	92.15	92.40
**Death Rate^b^**	**per 100,000 population**	
crude all cause	819.6	810.2
age-adjusted all cause	756.6	732.8
crude suicide	13.4	12.9
age-adjusted suicide	13.0	12.6
crude undetermined	1.9	1.5
age-adjusted undetermined	1.8	1.5

^a^ Centers for Disease Control and Prevention. CDC WONDER. Percentage distributions computed from Bridged-Race Population Estimates 1990–2014 Results. Available from: https://wonder.cdc.gov/controller/datarequest/D116;jsessionid=4A645106A7C749B49849AFCCF48C13FB

^b^ Centers for Disease Control and Prevention. CDC WONDER. About Underlying Cause of Death, 1999–2015. Available from: https://wonder.cdc.gov/ucd-icd10.html

^c^ Percentages were annualized for 2011–2013

### Individual-level variables from NVDRS

Manner of death (suicide versus undetermined) was the outcome variable. Individual-level predictors were overtness of selected injury mechanisms/causes (gunshot and hanging/ suffocation versus drug intoxication); suicide note (yes versus no or unknown); prior suicide attempt (yes versus no or unknown); and primary mental diagnosis (depression or bipolar disorder versus none or unknown). Other individual-level covariates were autopsy status; current mental health treatment; crisis in past two weeks; number of intimate partner or legal or job or financial or school problems; blood alcohol concentration (grams per deciliter); number of other specified drug positives; physical health problem; age; sex; race/ethnicity; marital status; and education.

### County-level covariates and data sources

Linked to the microdata, there were two county-level medicolegal covariates: selection mode (elected or appointed) of the chief medical examiner or coroner and accreditation status/type of medicolegal death investigation system (accredited medical examiner, accredited coroner, unaccredited medical examiner, unaccredited coroner). The data source for selection mode was a CDC website [37], and those for the investigation systems were the respective websites of the two relevant accrediting agencies, the National Association of Medical Examiners [38] and the International Association of Coroners & Medical Examiners [39], and email or telephone communication with state, district, or county offices to resolve outstanding questions. A third county-level covariate was urbanicity, a surrogate for external forces that may support or inhibit medicolegal death investigations [40]. Operationalized across five categories (large metropolitan, small metropolitan, adjacent metropolitan, micropolitan or adjacent, rural) representing the 12 ordinal categories of the 2013 Urban Influence Codes, its data source was the County Area Health Resource File for 2014–2015 [41].

### Statistical analysis and hypotheses

Pursuant to profiling suicides and undetermined cases, we used a generalized linear mixed model (GLMM) to test the respective hypotheses that: among the pooled cases, the designation of a “suicide” manner of death would be more likely when (1) the injury mechanism/cause was gunshot or hanging/suffocation (as more overtly violent methods of suicide), versus drug intoxication; and there was documentation of (2) a suicide note, (3) a prior suicide attempt, or (4) a primary diagnosis of depression or bipolar disorder. Then we tested our final hypothesis that any observed associations, pertaining to hypotheses 2 through 4, were stronger among drug intoxication than gunshot/hanging cases. GLMM is a two-level model, which was logistic at the individual level and linear at the county level. We included a state-level random effect to incorporate the data structure of counties nested in a state and individuals nested in a county. The statistical software was SAS for Windows, version 9.4 (Cary, NC: SAS Institute Inc., 2002–2010).

## Results

There were 36,190 suicides and 4,391 undetermined deaths in the 17 NVDRS states between 2011 and 2013 (Table 2). Thirty-one percent of the suicides were accompanied by a suicide note, compared with 1.5% for the undetermined cases. Prevalence of documented suicide attempts was 18% among suicides and 1.5% among the undetermineds, with corresponding prevalences of comorbid depression and bipolar disorder of 28%, 23%, 4%, and 5%. Gunshot/hangings constituted 68% of the suicides, compared with 11% for the drug intoxication cases, whereas the latter constituted 55% of the undetermined cases versus 4% of the gunshot/hangings.

**Table 2:** Profiling suicide and undetermined death cases by demographics, precipitating circumstances, and investigation characteristics: 17 National Violent Death Reporting System states, 2011–2013.

	Manner of Death (%)			Manner of Death (%)			Manner of Death (%)	
Demographics	Suicide (n = 36,190)	Undetermined (n = 4,391)	Circumstances	Suicide (n = 36,190)	Undetermined (n = 4,391)	Investigation	Suicide (n = 36,190)	Undetermined (n = 4,391)
**Age (years)**	** **		**Prior suicide attempt**	** **		**Chief medical examiner/coroner**	** **	
15–34	28.5%	32.0	yes	18.0%	12.5	elected	37.7%	18.7
35–54	38.9	45.4	no/unknown	82.0	87.5	appointed	62.3	81.3
55–74	25.3	19.7	**Mental diagnosis**			**System accreditation/type**		
75+	7.2	2.9	depression	28.4	23.2	accredited coroner	5.2	4.5
**Sex**			anxiety disorder	1.7	2.7	medical examiner	25.2	45.0
male	77.8	61.7	bipolar disorder	3.8	4.8	unaccredited coroner	32.4	14.2
female	22.2	38.3	other	2.3	2.6	medical examiner	37.2	36.3
**Race/ethnicity**			no/unknown	63.8	66.7	**Mechanism/cause**		
White non-Hispanic	84.4	79.1	**Mental treatment**			gunshot/hanging	68.1	4.3
Black non-Hispanic	5.7	7.4	yes	29.7	32.6	drug intoxication	11.5	55.3
Hispanic	3.4	4.2	no/unknown	70.3	67.4	specified other	9.3	8.2
Other	6.5	9.3	**Recent crisis**			unspecified	11.0	32.1
**Marital status**			yes	10.4	6.2	**Suicide note**		
single	33.4	40.9	no/unknown	89.6	93.8	yes	30.9	1.5
married	35.3	24.8	**Personal problem**	** **		no/unknown	69.1	98.5
widowed/divorced/separated	30.0	31.6	0/unknown	55.2	82.4	**Autopsy**		
unknown	1.3	2.7	1	31.5	14.2	yes	51.2	83.1
**Education (years)**			2	10.3	2.8	no/unknown	48.8	16.9
0–8	3.3	3.0	3+	3.0	0.6	**Blood alcohol concentration**		
9–12	4.9	7.8	**Physical health problem**			0.00 grams/deciliter	32.9	44.7
13+	76.3	78.0	yes	19.2	21.1	0.01–0.07	5.7	8.8
unknown	15.6	11.2	no/unknown	80.8	78.9	> = 0.08	15.1	16.8
**County urbanicity**						no test/unknown	46.4	29.7
large metropolitan	42.6	57.6				**Other drug positive**		
small metropolitan	36.0	27.3				0/unknown	74.6	30.4
adjacent metrometropolitan	17.5	12.3				1	15.0	29.4
adjacent micropolitan	1.8	1.7				2	6.8	24.8
rural	2.0	1.1				3+	3.6	15.5

The multilevel, multivariable analysis showed that gunshot/hanging deaths were 41 times more likely than drug intoxication deaths to be classified by medical examiners and coroners as suicides (Table 3)—affirming the hypothesized association regarding overtness of injury mechanism/cause. Odds also were higher for the residual specified group, whose injury mechanisms included gassing, other poisoning, cutting, jumping (falling), and drowning (immersion). Consistent with our second hypothesis, cases with a note were 34 times more likely than cases with no note or unknown note status to be classified as suicide. Also affirmed were our third and fourth hypotheses, which concerned evidence of a prior suicide attempt or a primary diagnosis of depression or bipolar disorder, respectively. Decedents who had a prior suicide attempt were 2.4 times more likely than decedents without such history to be classified as suicide, and those with documented unipolar depression or bipolar disorder were respectively 61% and 41% more likely than their referent to be so classified.

**Table 3:** Adjusted odds ratios^a^ and 95% confidence intervals for suicide versus undetermined manner of death classification according to injury mechanism/cause and documentation of a suicide note and other selected psychological/psychiatric characteristics: 17 National Violent Death Reporting System states, 2011–2013.

	Odds Ratio (95% CI)	n
**Mechanism/cause**		
**gunshot/hanging**	41.14 (34.43, 49.15)^d^	24,848
**specified other**	3.60 (3.08, 4.21)^d^	2,642
**unspecified**	0.40 (0.35, 0.46)^d^	5,389
**drug intoxication**	1.00	7,702
**Suicide note**		
**yes**	33.90 (26.11, 44.05)^d^	11,263
**no/unknown**	1.00	29,318
**Prior suicide attempt**		
**yes**	2.42 (2.11, 2.77)^d^	7,045
**no/unknown**	1.00	33,536
**Mental diagnosis**		
**depression**	1.61 (1.38, 1.88)^d^	11,295
**bipolar disorder**	1.41 (1.10, 1.81)^c^	1,575
**anxiety disorder**	0.83 (0.60, 1.16)	718
**other**	1.39 (1.03, 1.86)^b^	966
**no/unknown**	1.00	26,027

^a^ Adjusted for autopsy status, blood alcohol concentration, specification of one or more drugs, selection mode of chief medical examiner/coroner, accreditation status/type of medicolegal death investigation system, mental treatment, recent crisis, number of personal problems, physical problem, age, sex, race/ethnicity, marital status, education, and county urbanicity.

^b^ p < 0.05

^c^ p < 0.01

^d^ p < 0.001

### Stratification by mechanism/cause

Focus on a comparison of drug intoxication and gunshot/hanging suicides revealed marked differentials in their respective prevalences of a documented suicide note, prior suicide attempt, and a primary diagnosis of depression and bipolar disorder (Table 4). Prevalence gaps for a suicide note, prior suicide attempt, and affective disorders between suicides and undetermined deaths were larger among drug intoxication cases than gunshot/hanging cases. Multilevel, multivariable analysis affirmed our fifth and final hypothesis that observed associations between psychological and psychiatric characteristics and a suicide versus undetermined classification were stronger among the drug intoxication cases (Table 5). Relative to their referent, cases with a note were associated with 45-fold increased odds of a suicide classification for drug deaths compared with 8-fold increased odds for gunshot/hanging deaths. Documentation of a prior suicide attempt and comorbid depression showed increased odds of suicide classification of 164% and 48%, respectively, for the drug cases, but no associations for gunshot/hanging cases.

**Table 4:** Prevalence of a suicide note and other selected psychological/psychiatric characteristics documented in drug intoxication and gunshot/hanging suicide and undetermined death cases: 17 National Violent Death Reporting System states, 2011–2013.

	Drug intoxication			Gunshot/Hanging	
	Suicide (n = 4,175)	Undetermined (n = 2,430)		Suicide (n = 24,657)	Undetermined (n = 191)
**Suicide note**			**Suicide note**		
yes	42.2%	1.3%	yes	29.4%	4.2%
no/unknown	57.8	98.7	no/unknown	70.6	95.8
**Prior suicide attempt**			**Prior suicide attempt**		
yes	34.5	14.0	yes	14.9	10.0
no/unknown	65.5	86.0	no/unknown	85.1	90.0
**Mental diagnosis**			**Mental diagnosis**		
depression	43.6	26.8	depression	26.6	13.1
bipolar disorder	7.6	5.2	bipolar disorder	3.0	3.7
anxiety disorder	2.5	2.7	anxiety disorder	1.6	0.5
other	2.6	2.1	other	2.1	1.0
no/unknown	43.7	63.2	no/unknown	66.8	81.7

**Table 5:** Adjusted odds ratios^a^ and 95% confidence intervals for suicide versus undetermined manner of death classification by injury mechanism/cause according to documentation of a suicide note and other selected psychological/psychiatric characteristics: 17 National Violent Death Reporting System states, 2011–2013.

	Mechanism/Cause			
	Drug intoxication		Gunshot/Hanging	
Suicide note	Odds Ratio (95% CI)	n	Odds Ratio (95% CI)	n
yes	45.43 (31.06, 66.58)^c^	1,792	8.06 (3.94, 16.49)^c^	7,244
no/unknown	1.00	4,813	1.00	17,604
**Prior suicide attempt**				
yes	2.64 (2.19, 3.18)^c^	1,782	1.32 (0.79, 2.19)	3,680
no/unknown	1.00	4,823	1.00	21,168
**Mental diagnosis**				
depression	1.48 (1.17, 1.87)^b^	2,470	1.26 (0.74, 2.15)	6,579
bipolar disorder	1.39 (0.98, 1.97)	445	0.61 (0.26, 1.39)	753
anxiety disorder	0.62 (0.38, 1.00)	171	2.20 (0.30, 16.41)	385
other	1.54 (0.93, 2.55)	157	1.68 (0.40, 7.10)	517
no/unknown	1.00	3,362	1.00	16,614

^a^ Adjusted for autopsy status, blood alcohol concentration, specification of one or more drugs, selection mode of chief medical examiner/coroner, accreditation status/type of medicolegal death investigation system, mental treatment, recent crisis, number of personal problems, physical problem, age, sex, race/ethnicity, marital status, education, and county urbanicity.

^b^ p < 0.01

^c^ p < 0.001

## Discussion

Even the enriched microdata from the NVDRS showed less than one-third of suicide cases included a record of a comorbid affective disorder, prior suicide attempt, or authenticated suicide note, whether written, typed, digital or audio. Signaling complications for suicide accounting and prevention, our multilevel, multivariable analysis leads us to infer that full and accurate accounting of suicide by opioid and other drug intoxication in the US is far more dependent upon corroborative psychological and psychiatric evidence than is that of suicide by the other two major but overtly violent methods, firearm and hanging/suffocation. We hypothesize that absence of such evidence increases the likelihood that drug intoxication suicides are misclassified in the accident manner of death category, as well as in undetermined. Against the background of highly stressed emergency healthcare [7,8] and medicolegal death investigation systems [9,10], our findings both reinforce and inform concern and skepticism about the uniformity and validity of suicide and default manner of death classifications in the US [32,42–44].

During the late 1980s, CDC convened an expert multidisciplinary panel to develop operational criteria for suicide determination [45]. This panel recommended that injury deaths be classified as suicide if the injury was consistent with self-infliction and there was indication of intent. They also recommended medical examiners and coroners utilize multiple data sources in their investigations, including sources that would facilitate identification of the psychological and psychiatric characteristics of suicides and possible suicides. In cases where intent was equivocal, medical examiners and coroners would need to consult psychiatrists or clinical psychologists proficient in assessing self-harm proximal to death in order to optimize suicide ascertainment [13]. However, there continues to be no such auxiliary workforce in the US to support suicide investigations, unlike police in homicide investigations, whose training and service focus on ruling homicide in or out in death scene and other medicolegal death investigations [46]. Moreover, medical examiners and coroners rarely employ any formal psychological inquiry in helping them resolve intentionality or manner of death in ambiguous, uncertain, and equivocal cases, epitomized by most drug poisoning deaths [11,47]. Such procedures, in some settings called a “psychological autopsy,” integrate interviews with key informants—family, friends, and acquaintances of decedents—and intensive records review and examination [17,48].

Heterogeneity of forensic investigation systems, within and across states, may impede accurate classification of suicide and other manners of death in the US [31,49]. With no national oversight, administration of death investigation systems varies from state control under an appointed state medical examiner to county control under an elected coroner or a medical examiner [50]. Contrasting with medical examiners, coroners typically lack training in medicine, pathology or forensic pathology. Training in psychiatry or clinical psychology is not a requisite for either position. Compounding threats to the quality of injury-related manner of death data, most coroner offices were not professionally accredited by either the International Association of Coroners & Medical Examiners or the National Association of Medical Examiners, as of January 2015 [51].

In 2015, CDC hosted an expert meeting to commence addressing the inherent challenges fatal opioid and other drug intoxication cases pose for medical examiner and coroners in accurately assigning suicide and other manners of death [52]. Our research will fortify the empirical underpinnings of this important and ongoing initiative.

### Strengths and limitations

The first multilevel investigation of the association between psychological/psychiatric documentation and suicide manner of death classification in the US, and in the world to the best of our knowledge, this study was able to factor in heterogeneity across precipitating circumstances of death, medicolegal death investigation systems and practices, and demographics. Only the microdata from the NVDRS Restricted Access Database were appropriate for evaluating our research questions. Essential for valid geographic, sociodemographic, and temporal accounting and comparisons, the NVDRS emphasizes uniform protocols for defining manner of death and achieving consistent data collection, entry, review, and coding practices [33]. However, the affirmation of hypothesized variation between suicides and undetermined deaths is likely conservative as a consequence. Tempering this caveat on study generalizability is the countervailing demographic concordance we reported between the 17 NVDRS states and the nation. We note that since our observation period, 2011–2013, the NVDRS has expanded to 40 states, the District of Columbia, and Puerto Rico [1]. The goal is to cover all 50 states and territories.

NVDRS data do not include staffing and budgetary information on medicolegal offices, and data collection is not yet uniform and standardized for mental health, substance use, and other circumstances of death. Toxicology is nearly complete in drug and other poisoning cases, but fiscal constraints and associated data deficits make coverage problematic for surveillance and research in non-poisoning cases [53]. The “yes” provision means affirmative for circumstances of the suicide and undetermined deaths, but “no” entries are not distinguished from unknown or missing information—a conservative bias. The NVDRS neither documents comorbid diseases, such as cancer and amyotrophic lateral sclerosis (ALS), which are risk factors for suicide [54], nor differentiates prescription opioids from illicit analogs. Data sparseness in our multilevel study precluded more refined distinctions among injury mechanisms/causes and separation of centralized and decentralized medicolegal death investigation systems.

## Conclusions

Without psychological/psychiatric evidence contributing to manner of death classification, suicide by drug intoxication in the US is likely profoundly under-reported. Findings harbor adverse implications for surveillance, etiologic understanding, and prevention of suicides and drug deaths.
